# 142. When You Can't Call It Colonization, Consider Cutting It Out: Mitigating Panels of Unusual Size to Improve C. difficile Diagnostic Specificity

**DOI:** 10.1093/ofid/ofad500.215

**Published:** 2023-11-27

**Authors:** Dan Ilges, Leah Grant, Angela Huang, Eric Siebeneck, John Castro, Maria T Seville, Thomas Grys, Lisa Speiser, Erin Graf

**Affiliations:** Mayo Clinic Arizona, Phoenix, Arizona; Mayo Clinic Arizona, Phoenix, Arizona; Mayo Clinic Arizona, Phoenix, Arizona; Mayo Clinic Arizona, Phoenix, Arizona; Mayo Clinic Arizona, Phoenix, Arizona; Mayo Clinic Arizona, Phoenix, Arizona; Mayo Clinic Arizona, Phoenix, Arizona; Mayo Clinic in Arizona, Phoenix, Arizona; Mayo Clinic Arizona, Phoenix, Arizona

## Abstract

**Background:**

The BioFire® FilmArray® Gastrointestinal Pathogen Panel (GIPP) tests for 22 potential pathogens in stool, including Clostridioides difficile. C. difficile nucleic acid is detected via nested polymerase chain reaction (PCR) targeting toxin A (TcdA) and/or toxin B (TcdB) genes. While PCR-based diagnostic methods are highly sensitive, they lack specificity to differentiate colonization from C. difficile infection (CDI). Mayo Clinic Arizona (MCA) uses enzyme immunoassay (EIA) testing for toxin A/B and glutamate dehydrogenase antigens with reflex to PCR for discordance as the primary algorithm for diagnosing CDI; however, GIPP PCR testing circumvents this algorithm. When used in patients with low pretest probability for CDI, clinical false positives can lead to inappropriate diagnosis and treatment of colonization.

GIPA Results in the Electronic Medical Record Pre-Intervention

**Figure:**
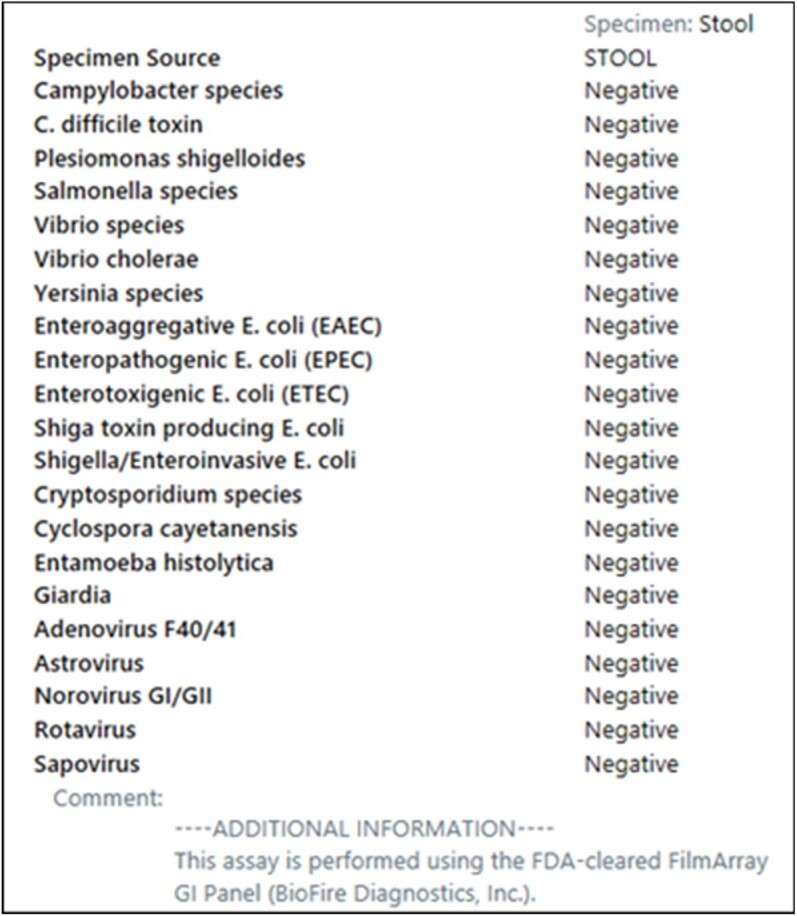

MCA Standalone C. difficile Testing Algorithm

**Figure:**
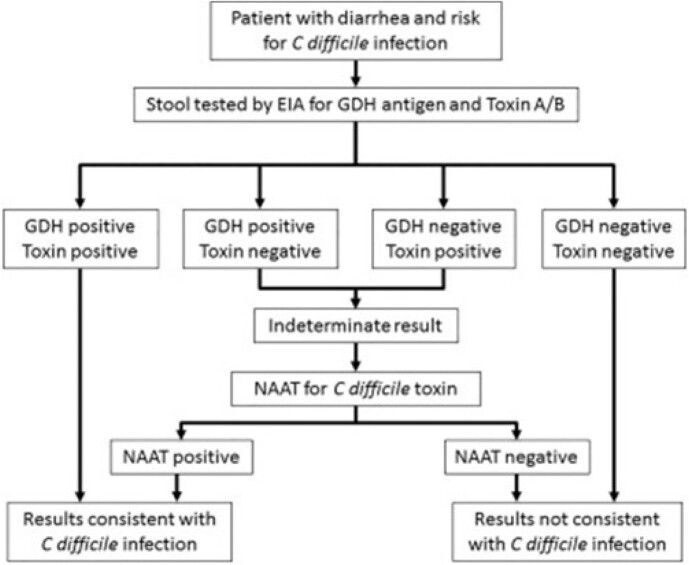

Abbreviations – EIA, enzyme immunoassay; GDH, glutamate dehydrogenase; NAAT, nucleic acid amplification test

**Methods:**

We convened a multidisciplinary quality improvement team to reduce the detection and treatment of C. difficile colonization secondary to GIPP test results. Surveys were sent to providers and nurses as part of a formal gap analysis and included questions around ordering patterns, knowledge of appropriate tests, and associated costs. Baseline data was compiled from GIPP orders in MCA from 1/1/2022-9/30/2022.

**Results:**

There were 1978 GIPP orders for 1694 patients during the study period (∼7 GIPPs/day). Most orders (65%) originated from ambulatory settings. The most common positive target was C. difficile (355/723, 49%), which resulted in treatment for suspected CDI in 94.9% (337/355) of cases. Of the 49 providers who responded to the survey, 86% ordered the GIPP at least monthly. Testing for C. difficile was the 2nd most common reason for ordering the GIPP. Most providers underestimated the cost of the panel and were unfamiliar with the differences between the GIPP and EIA testing algorithm. Nurses sought clarification on stool samples suitable for GIPP testing. Following education and modifications to ordering in the electronic health record, the decision was made to remove the C. difficile target from the GIPP.

Provider Understanding of C. difficile Testing Methods

**Figure:**
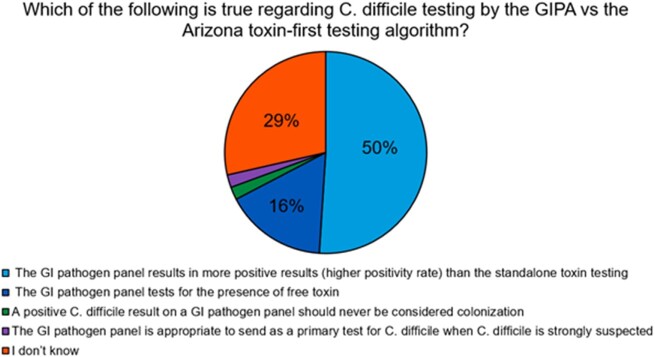

GIPA Results in the Electronic Medical Record Post-Intervention

**Figure:**
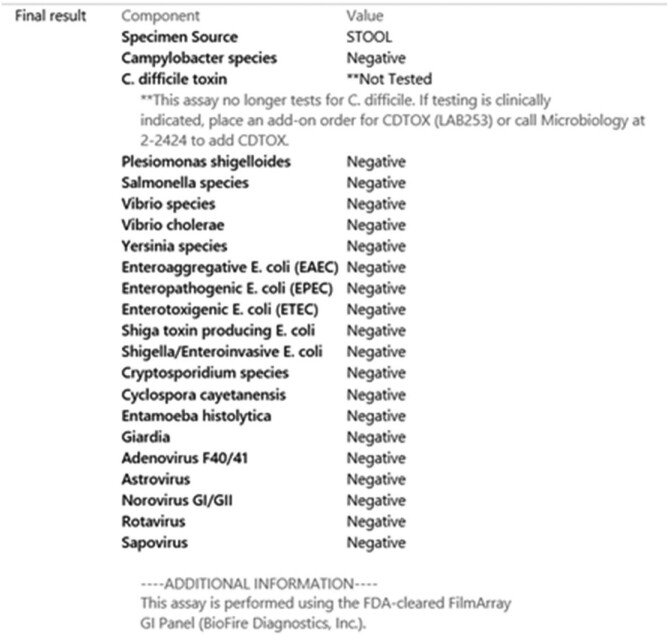

**Conclusion:**

Multiplex PCR panels are comprehensive but require careful implementation to facilitate diagnostic stewardship. Facilities with robust standalone C. difficile testing algorithms may consider removing the C. difficile target from the GIPP.

**Disclosures:**

**All Authors**: No reported disclosures

